# Neural responses reveal associations between personal values and value-based decisions

**DOI:** 10.1093/scan/nsaa150

**Published:** 2020-11-04

**Authors:** Yun-Shiuan Chuang, Yu-Shiang Su, Joshua O S Goh

**Affiliations:** Department of Psychology, National Taiwan University, Taipei 10617, Taiwan; Graduate Institute of Brain and Mind Sciences, College of Medicine, National Taiwan University, Taipei 10051, Taiwan; Neurobiology and Cognitive Science Center, National Taiwan University, Taipei 10617, Taiwan; Graduate Institute of Brain and Mind Sciences, College of Medicine, National Taiwan University, Taipei 10051, Taiwan; Taiwan International Graduate Program in Interdisciplinary Neuroscience, National Taiwan University and Academia Sinica, Taipei 10617, Taiwan; Department of Psychology, National Taiwan University, Taipei 10617, Taiwan; Graduate Institute of Brain and Mind Sciences, College of Medicine, National Taiwan University, Taipei 10051, Taiwan; Neurobiology and Cognitive Science Center, National Taiwan University, Taipei 10617, Taiwan; Center for Artificial Intelligence and Advanced Robotics, National Taiwan University, Taipei 10617, Taiwan

**Keywords:** personal values, Schwartz Value Survey, value-based decision-making, fMRI, motivation

## Abstract

Personal values are thought to modulate value-based decisions, but the neural mechanisms underlying this influence remain unclear. Using a Lottery Choice Task functional brain imaging experiment, we examined the associations between personal value for hedonism and security (based on the Schwartz Value Survey) and subjective neurocognitive processing of reward and loss probability and magnitude objectively coded in stimuli. Hedonistic individuals accepted more losing stakes and showed increased right dorsolateral prefrontal and striatal and left parietal responses with increasing probability of losing. Individuals prioritizing security rejected more stakes and showed reduced right inferior frontal and amygdala responses with increasing stake magnitude, but increased precuneus responses for high-magnitude high-winning probability. With higher hedonism, task-related functional connectivity with the whole brain was higher in right insula and lower in bilateral habenula. For those with higher security ratings, whole-brain functional connectivity was higher in bilateral insula, supplementary motor areas, right superior frontal gyrus, dorsal anterior cingulate cortex, and lower in right middle occipital gyrus. These findings highlight distinct neural engagement across brain systems involved in reward and affective processing, and cognitive control that subserves how individual differences in personal value for gaining rewards or maintaining *status quo* modulate value-based decisions

## Introduction

An offer of $100 in a stake that comes with a 60% chance for penalty of that same amount might be accepted by some persons more so than others (Kahneman and Tversky, 1979; Schunk and Betsch, 2006). Such findings motivate the need for understanding how the brain often yields different decision behaviors across persons in response to identical choice stimuli (Kable and Glimcher, 2007; Crapse and Basso, 2015; Kable and Levy, 2015). Variability in decision behaviors has been associated with differences in personal values, which are abstract subjective beliefs or ideals about what actions and outcomes are congruent with an individual’s goals or self-identity (Feather, 1995). In the present study, we evaluated how differences in personal value for gaining rewards or maintaining *status quo* in life modulate neural processing of stimuli with economic-related information and the accompanying differences in risk acceptance or avoidance behavior. To this end, we assessed the degree individuals valued hedonism and security, which are personal value constructs from the Schwartz Value Survey (SVS; Schwartz, 1992 1994) that we considered relevant to economic value-based decision-making. We then examined the associations between subscription to these personal values and neural and behavioral responses to potential rewards (and losses) through a functional magnetic resonance imaging (fMRI) Lottery Choice Task (LCT) experiment.

In the LCT, participants accept or reject trials with different expected values (EVs) constituted by different probabilities of winning different magnitudes of points (Goh *et al.*, 2016; Su *et al.*, 2018). In principle, gains in the LCT are maximized by accepting positive EV trials and rejecting negative EV trials (see the ‘Methods’ section). Deviation from this decision heuristic suggests subjectively biased underlying neurocognitive processes operating on the objective reward information conveyed in the stimuli. Studies show that stimuli associated with higher levels of expectation of reward engage higher ventral striatal and prefrontal activity (Knutson *et al.*, 2001 2003 2005; Camerer, 2008; Rangel *et al.*, 2008; Haber and Knutson, 2010; Dolan and Dayan, 2013). Using the LCT, we previously found that individual risk preferences further modulated such reward-related responses in these brain areas amongst others (Goh *et al.*, 2016). Whereas medial temporal, ventromedial frontal and ventral striatal activity generally increased with ‘increasing’ EV in risk-averters, in line with previous studies, neural activity increased with ‘decreasing’ EV in these brain areas as well as in lateral frontal areas in risk-takers. Such lateral frontal involvement in risk-takers for expected losses in the LCT may reflect regulatory control of negative affect to license behavioral acceptance of losing stakes (Dodds *et al.*, 2011; Shenhav *et al.*, 2013), although this remains speculative. At the very least, systemic functional neural responses during value-based decision-making are indicative of subjective reward processing variability that might be associated with individual differences in personal values.

Of relevance to the present study on value-based decision processing, the personal value of hedonism is characterized in the SVS by an emphasis on reward and pleasure, whereas security emphasizes certainty and stability (Schwartz, 1992 1994). In the context of LCT performance, we reasoned that higher hedonism indicates a preference for prospective gains and downplaying of potential losses. As such, higher hedonism should be associated with higher acceptance of losing stakes, where the potential for winning is present but the probability of losing is high. In contrast, higher prioritizing of security indicates a preference for options that preserve the *status quo* or guarantee prospects. Thus, individuals prioritizing security should show a propensity to reject stakes in the LCT in order to minimally affect current gains and would risk accepting potential gains mostly when stake magnitudes are low or when winning probabilities are high.

Critically, we were interested in whether individuals who differentially subscribed to hedonism and security would evince distinct neural responses to stimuli coding variable probabilities and magnitudes of reward. Because hedonism entails pursuit of rewards, we considered it reasonable to expect individual differences in hedonism to be associated with response modulation in reward-related areas including ventral striatum and frontal cortex when processing an anticipated stimuli value. In particular, if indeed hedonistic individuals subjectively emphasize potential rewards even when losses are likely, reward-related brain areas should evince higher responses to losing stakes in those with higher hedonism compared to their counterparts, particularly when stake magnitudes are high. In contrast, we reasoned that when individuals who emphasize security accept low-risk stakes, such decisions might be motivated more by a need to hedge one’s status than pursuit of reward. As such, neural responses to stimuli value in brain areas other than the above reward-related areas should evince associations with individual differences in subscription to security. Finally, we considered that hedonism and security are complex constructs involving distributed neural network processing apart from regional neural activation levels. Thus, we also evaluated how these personal values are associated with functional degree centrality during LCT performance as a measure of task-related communication between a given brain area and the rest of the brain neural network (Buckner *et al.*, 2009; Rubinov and Sporns, 2010).

## Methods

### Participants

Forty-four right-handed participants from the local community were recruited for this study. Exclusion criteria included presence or history of neurological or psychiatric diseases and contraindications for MRI scanning. All participants were remunerated for time spent and provided written informed consent for this study, which was approved by the National Taiwan University Hospital Research Ethics Committee. One participant opted out during follow-up neuropsychological testing. Data for the remaining 43 participants (mean age = 23.75 years, s.d. = 2.02 years, range = 20.16−28.53 years; 17 males, 26 females) were entered into the behavioral analysis. Three out of the 43 participants had excessive head motion during MRI scanning (> 2 mm translation or > 2° rigid-body rotation) and therefore fMRI data analyses were conducted in the remaining 40 participants (mean age = 23.65 years, s.d. = 1.97 years, age range = 20.16−28.53; 14 males, 26 females). Note that the present study extends our previous analysis of the same 40 young adult data (Su *et al.*, 2018) by specifically and more comprehensively considering the involvement of personal values. Sample size and power considerations are detailed in the Supplementary Methods.

## Materials and procedures

### Lottery Choice Task

**Fig. 1.**
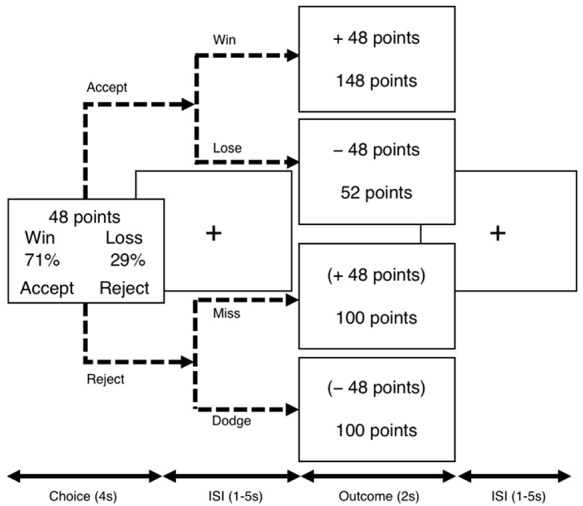
Procedure of the Lottery Choice Task (LCT). During the choice phase, the winning probability (*P*) and magnitude (*M*) of the stake were explicitly presented for 4 s on the screen for participants’ decision. Participants were tasked to either accept or reject the trial. The example above depicts a trial in which there is a 71% chance to win 48 points, which is also a 29% chance to lose 48 points. After inter-stimuli intervals (ISIs) ranging from 1 s to 5 s, the outcome phase presented the result of the lottery and the total accumulated points for 2 s. If participants chose to accept the lottery trial, the outcome phase showed either gain or loss with increased or decreased accumulated points, respectively. Rejected trials yielded outcomes as well but within parentheses, with unchanged accumulated points. The next trial then began after another intervening ISI.

There were 225 LCT trials, each with a choice and outcome phase, in this event-related fMRI experiment (Figure 1; see Goh *et al.*, 2016). During the choice phase, numerical text stimuli depicted the magnitude of points at stake (*M*) and the percentage of winning probability (*P*), which was simultaneously the alternative losing probability (1−*P*) of the given stake. *P* and *M* constituted EV = *P* × *M* + (1−*P*) × (−*M*) of each trial. Numerical values of probability and magnitude varied continuously over trials. To facilitate even distribution of choice stimuli values and outcomes throughout the task, we discretized choice stimuli into five levels of winning (simultaneously losing) probability (LL: low-low, ML: middle-low, MM: middle-middle, MH: middle-high, HH: high-high) and three levels of point magnitudes (L: low, M: middle, H: high) that combined to yield 15 choice conditions (see Supplementary Table S1). Participants accepted or rejected the displayed stakes using assigned button presses to maximize points accumulated in the LCT. Choice stimuli remained on screen for a full 4 s within which participants had to respond.

The choice phase was followed by the outcome phase (2 s). Outcomes were stochastically predetermined based on the given *P* with the requirement that no choice condition could have only winning or losing outcomes. For accepted stakes, gain or loss outcome points and accumulated points would be displayed on screen. For rejected stakes, missed outcomes would be presented in parentheses, along with unchanged accumulated points. Null responses yielded zero-point outcomes along with a reminder message. Inter-stimuli intervals (ISIs) ranged from 1 s to 5 s and separated all choice and outcome stimuli. The mean (s.d.) ISI was 1.36 (0.87) s between choice and outcome phases and 1.44 (1.02) s between outcome phase of the previous trial and choice phase of the next trial.

The 15 choice conditions were pseudo-randomly ordered in each of the five fMRI runs with three trials per condition per run, no more than two consecutive trials of the same condition and no repeated combinations of probability and magnitude. LCT stimuli were presented using E-Prime software Version 2.0 (Psychological Software Tools, Sharpsburg, PA, USA). Stimuli in the scanner room were back-projected onto a screen at the front of the MRI bore viewed through a mirror mounted on the receiver head coil. Participants underwent a practice version outside of the scanner on a notebook to ensure they understood the rules and were familiar with the ranges of stimuli values prior to the actual fMRI experiment. The experiment was conducted in Chinese, the first language of all the participants.

### Schwartz Value Survey

Participants completed the Chinese version of the SVS (Schwartz, 1992; Schwartz and Sagiv, 1995) on a separate day during a neuropsychological test session within 1 month of the MRI scan. Participants indicated the degree of personal importance of each SVS item using a 9-point scale that ranged from 0 (*not at all important*) to 7 (*extremely important*), and −1 (*opposite to what I value*). Sub-value items for hedonism and security are listed in the Supplementary Methods. Cronbach’s alphas were 0.74 for hedonism and 0.75 for security (see Supplementary Figure S1 for rating distributions and details).

### Behavioral acceptance rate data analysis

Behavioral data analysis was implemented using R version 3.4.3 (R Development Core Team, 2017) with lme4 version 1.1-16 (Bates *et al.*, 2015) and lmerTest version 3.0.1 using Satterthwaite approximation for degrees of freedom (Kuznetsova *et al.*, 2017). Hedonism and security SVS ratings were centered by each participant’s own average whole-questionnaire item rating to adjust for potential participant bias in scale use (Schwartz, 2009). To evaluate the association between SVS personal values and LCT acceptance rates (AR = *N_accepted trials_*/*N_responded trials_*), all participant’s binary decision responses (1 = accept; 0 = reject) for all trials were entered as the dependent variable in the following mixed-effects logistic regression model (Jaeger, 2008):

(1) \begin{align*} log\, it(D) = &\, {b_o} + {b_{sex}}Sex + {b_H}H + {b_s}S + {b_p}P + {b_M}M + {b_{PM}}PM \nonumber\\ & + {b_{HP}}HP + {b_{SP}}SP + {b_{HM}}HM + {b_{SM}}SM + {b_{HPM}}HPM \nonumber\\ & + {b_{SPM}}SPM + {v_{oi}} + {v_{pi}}P + {v_{Mi}}M + {v_{pMi}}PM \end{align*}

In Equation 1, *D* denotes trial-wise binary decision responses, *S* and *H* are participant ratings for security and hedonism, respectively, and *P* and *M* are the trial-wise continuous winning probability and magnitude of points at stake, respectively. Each continuous predictor was z-transformed prior to composing interactive terms. *Sex* was included as a covariate (male = −1, female = 1). *b* denotes the fixed effect coefficients and *v_i_* the random effects coefficients for the *i*th participant. Thus, the above model estimated the fixed effects of security (*b_S_, b_SP_, b_SM_, b_SPM_*) and hedonism (*b_H_, b_HP_, b_HM_, b_HPM_*) on ARs over probabilities and magnitudes in the LCT each, while controlling for the other personal value and participant-specific responses to the LCT variables (Barr, 2013). Positive and negative coefficients indicate that the personal values were associated with increased and decreased ARs, respectively, with increasing probability and magnitude given the base AR (*b_0_*) and effects of probability and magnitude *(b_P_, b_M_, b_PM_*). Analysis of response time data is detailed in Supplementary Methods.

### Brain imaging protocol and preprocessing

Brain imaging data were acquired in a 3T Siemens Skyra MRI scanner located at the National Chengchi University, Taipei, Taiwan, using a 32-channel head coil. Image acquisition and preprocessing details have been reported by Su *et al*. (2018; see also Supplementary Methods). Briefly, each participant afforded (i) five runs of whole-brain functional images (218 volumes) using a gradient-echo echo planar imaging sequence with 38 axial slices, voxel size 3.4375 × 3.4375 × 4 mm and repetition time 2000 ms, (ii) a high-resolution T2 image coplanar to the functional scans for co-registration and (iii) a high-resolution whole-head structural T1 with 192 sagittal slices and voxel size 1 × 1 × 1 mm for normalization to Montreal Neurological Institute (MNI) template space. Preprocessing and analyses of functional brain data were conducted using SPM12 revision 6906 (Statistical Parametric Mapping, Wellcome Trust Centre for Neuroimaging, London, UK; http://www.fil.ion.ucl.ac.uk/spm/). Preprocessing steps included slice-timing and motion correction, spatial normalization to template space with resampling to 3 mm isovoxel size, and 3D spatial smoothing with an 8 mm Gaussian kernel.

### fMRI LCT neural response analysis

For each participant, first-level model-based fMRI analysis was conducted on the preprocessed functional images. We used a general linear model (GLM) with delta function regressors for the choice-phase onset (CHOICE) and three types of outcome-phase onsets (ACCEPT, REJECT and if present, NULL: missed choice-phase responses) convolved with the hemodynamic response function (HRF). Additional choice-phase regressors further parametrically modulated the HRF-convolved choice-phase onset regressor by trial-wise probability (PROB), magnitude (MAG), probability × magnitude (*P* × *M*) and current accumulated points (ACCUM) (see Supplementary Methods). Except for null outcomes, four outcome-phase regressors (WIN: accepted gains, LOSS: accepted losses, MISS: avoided gains, DODGE: avoided losses) that parametrically modulated the respective HRF-convolved outcome-phase onset regressors by the magnitude of gain or loss were included. Null outcome-phase responses were HRF-modeled as dummy responses without parametric modulation. Finally, six movement parameters were included as covariates (three translations, three rotations) along with a constant resulting in a total of 17 (18 if NULL existed) regressors per participant per run. Contrasts averaging the resulting model coefficients across the five runs then afforded whole-brain voxel-wise mean neural response estimates for the regressors. In this study, we focus on the effect of personal values on LCT variable representations during choice processing so outcome-related responses are not further analyzed. Overall, the regressors PROB, MAG and *P* × *M* yielded whole-brain voxel-wise parameter estimates for neural sensitivity to trial-wise changes in these respective LCT variables during choice processing for each participant. For example, higher positive voxel coefficients for PROB indexed greater positive neural sensitivity to trial-wise variations in stake-winning probability. For such voxels, blood-oxygen-level-dependent (BOLD) responses are higher for winning (*P* > 0.5) than losing (*P* < 0.5) probability. Similarly, lower (more negative) coefficients for PROB indexed greater negative neural sensitivity to stake-winning probability. For such voxels, BOLD responses are higher for losing than winning probability. Coefficients for PROB around zero indicate similar BOLD responses to winning and losing probability.

To evaluate brain areas in which personal values modulated neural sensitivity to the LCT variables, the above individual whole-brain parameter estimates were passed as dependent variables in group-level GLMs that included individual security and hedonism ratings as regressors and sex as a covariate. Whole-brain one-sample *t*-test contrasts then evaluated the regions showing significant effects of hedonism and security on voxel-wise neural sensitivity to the LCT variables. Whole-brain contrast statistical significance thresholds were applied using cluster-wise adjustment of family-wise error at *P*(FWE) < 0.05 as computed in Monte Carlo simulations with 10 000 iterations, separately for cortical and subcortical masked areas, using 3dClustSim in AFNI version 19.0.24 (Forman *et al.*, 1995; Cox *et al.*, 2017) (see Supplementary Methods for details).

Functional regions-of-interest (ROIs) were defined only to aid visualization of personal value association trends with neural responses in significant clusters from whole-brain contrasts, particularly the *P* × *M* interaction. ROIs were contiguously significant voxels within a sphere of 8 mm radius around peak contrast voxels from the second-level contrasts. Individual neural response parameter estimates for the variables PROB and *P* × *M* were extracted from each ROI from participants’ first-level contrasts and averaged across all voxels in the ROI. For each participant, conditional neural response sensitivity (CONDPROB) to probability under three different magnitude levels were then calculated for each ROI using the extracted PROB and *P* × *M* response estimates and each magnitude level mean such that CONDPROB = PROB + (*P* × *M*) × *magnitude level mean*.

### fMRI LCT degree centrality analysis

Functional brain degree centrality of each voxel is the average of the correlations between the functional time course of that voxel with the time courses of the rest of the brain gray matter voxels and reflects the degree of functional connectivity between a voxel and the whole brain. Preprocessing steps for degree centrality image analysis are detailed in the Supplementary Methods. Derived degree centrality images from all participants were submitted as the dependent variables in a univariate whole-brain voxel-wise GLM with individual hedonism and security ratings as regressors and sex as a covariate. Whole-brain contrast statistical significance was set at cluster-wise *P*(FWE)* *< 0.05 as above (see Supplementary Methods for details).

## Results

### LCT acceptance rate behavior

Effects of trial-wise differences in probability and magnitude on LCT decision behavior for the participants in this study have been reported by Su *et al.* (2018; young adult group). We describe these primary effects of probability and magnitude on ARs based on Equation 1 in the Supplementary Results for validation.

**Fig. 2.**
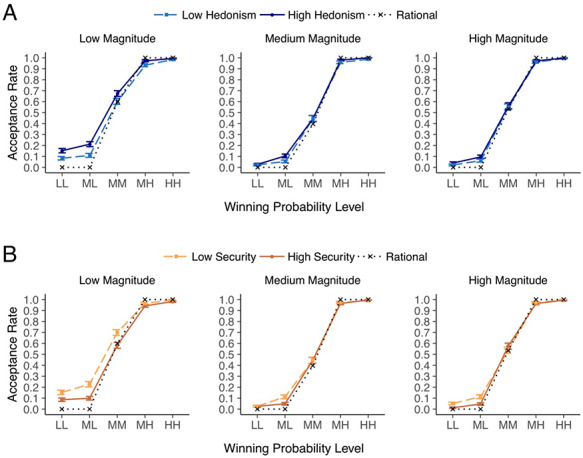
Associations between (A) hedonism and (B) security and acceptance rates (ARs) in the Lottery Choice Task (LCT). Participants’ mean ARs are shown over three magnitude levels and five winning probability levels based on a median split of the respective personal value ratings (see Supplementary Figure S1). LL = low-low; ML = middle-low; MM = middle-middle; MH = middle-high; HH = high-high. Error bars represent ±1 SE. The dotted line marks the trend of a hypothetical rational decision-maker who solely relies on the expected value (EV) of lottery trials (accept if EV > 0 and reject if EV < 0). Missed decisions [mean (s.d.) = 2.37 (3.34) per person] were not included in the figure. See Supplementary Figure S4 for modeled ARs based on coefficients estimated using Equation 1.

Here, we examined the effects of hedonism and security associated with ARs in Equation 1 (Supplementary Table S2). For hedonism, there was a significant hedonism × probability × magnitude interaction effect (H × *P* × *M: b *= 0.35, SE = 0.17, *z *= 2.06, *P *= 0.039, 95% confidence interval (95% CI) [0.02, 0.68]). There was no significant base effect of hedonism in increasing overall AR and no effect of hedonism on AR in interaction with probability or magnitude alone. Figure 2A visualizes mean ARs across high and low hedonism groups defined using a median split, across the three discrete magnitude levels and five probability levels (modeled trends are shown in Supplementary Figure S4A). As can be seen, higher hedonism ratings were associated with increased stake acceptances as magnitudes and winning probabilities decreased.

Individuals with higher security ratings had lower mean ARs (S: *b* = −0.38, SE = 0.18*, z *= −2.14, *P *= 0.032, 95% CI = [−0.73, −0.03]) (Supplementary Table S2, Figure 2B, and Supplementary Figure S4B). Although we note the trend for associations with security to be accentuated at lower magnitudes, these interactions involving magnitude did not reach significance. Thus, individuals with higher security ratings rejected more trials (and faster; see Supplementary Results) than those with lower ratings.

### Hedonism and security differentially modulate neural sensitivity to probability and magnitude

**Fig. 3.**
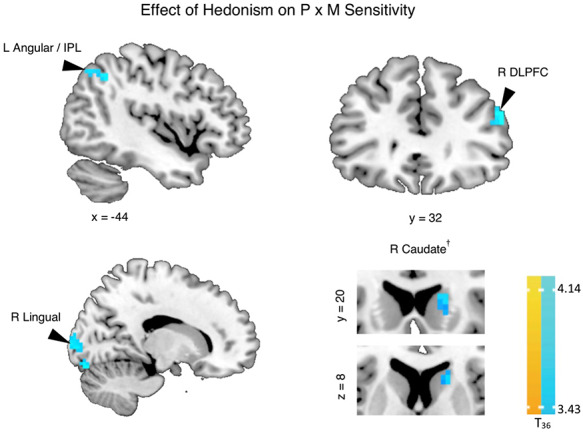
Whole-brain associations between higher hedonism personal value and neural sensitivity to value parameters during the Lottery Choice Task (LCT). Individuals with higher hedonism showed more negative neural sensitivity to the interaction between probability and magnitude (cool colors) such that there was higher neural sensitivity to increasing losing probability as magnitude value increased during the choice phase (see Supplementary Figure S6A, Supplementary Table S4). *P* × *M* = probability × magnitude; L = left; R = right; IPL = Inferior Parietal Lobule; DLPFC = dorsolateral prefrontal cortex. Contrast threshold significance level was set at cluster-wise *P*(FWE) < 0.05 (see the ‘Methods’ section). Whole-brain statistical overlay results are displayed on a standard anatomical MNI template. **^†^**: The results in the subcortical mask were derived from small volume correction with a primary voxel threshold of *P *< 0.005 (uncorrected) (see Supplementary Methods). Note that if the primary voxel threshold was set to *P *< 0.001 (uncorrected), the cluster threshold size would be *k* = 5, while the cluster size of caudate would shrink to *k* = 2 and become non-significant.

Effects of trial-wise variations in probability and magnitude on neural responses have been reported by Su *et al.* (2018; young adult group). Briefly, increasing winning probability, as well as increasing stake magnitude, was associated with linear increases in neural activity (positive neural sensitivity to EV) in ventral striatal (VS) regions and right inferior frontal gyrus (IFG). Critically, here, we found several frontoparietal and subcortical regions further showing associations between hedonism and the interactive influence between probability and magnitude (*P* × *M*) on neural responses (Figure 3, Supplementary Table S4).

Individuals with higher hedonism ratings evinced higher neural sensitivity to increasing losing probabilities that increased with stake magnitudes in the left angular gyrus/inferior parietal lobule (IPL), right dorsolateral prefrontal cortex (DLPFC) and caudate, as well as right lingual areas. No other significant whole-brain effects of hedonism were observed. To better visualize the trends underlying these whole-brain *P* × *M* responses, Supplementary Figure S6A plots the associations between hedonism and neural sensitivity to probability over low, middle and high levels of stake magnitudes in functional ROIs defined from the whole-brain contrast. Across all ROIs, the plotted trends illustrate that individuals with higher hedonism engaged lower (more negative) to higher (more positive) neural sensitivities to winning probability for high to low stake magnitudes, respectively. That is, BOLD responses in these ROIs for these participants were higher for losing than winning probability at higher than lower magnitudes. On the other hand, individuals with lower hedonism engaged higher to lower neural sensitivities to win probability from high to low magnitudes, respectively.

**Fig. 4.**
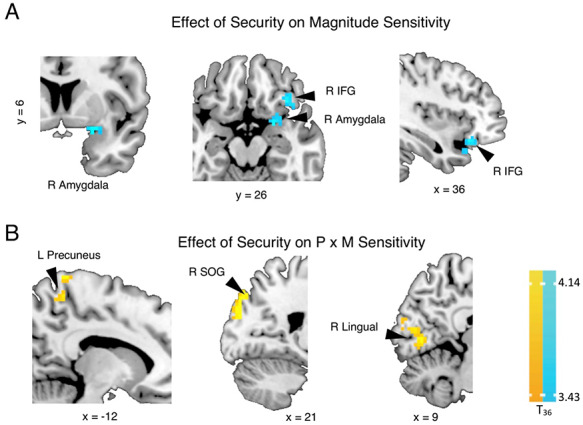
Whole-brain associations between higher security personal value and neural sensitivity to value parameters during the Lottery Choice Task (LCT). During the choice phase of the LCT, individuals with higher security value showed (A) higher neural sensitivity to decreasing magnitude (cool colors) across right inferior frontal and amygdala. Individuals with higher security value also showed (B) greater neural sensitivity to the positive interactive effect of probability and magnitude (warm colors) (see Supplementary Table S5, Supplementary Figure S6B). *P* × *M* = probability × magnitude; L = left; R = right; IFG = inferior frontal gyrus; SOG = superior occipital gyrus. Contrast threshold significance level was set at cluster-wise *P*(FWE) < 0.05 (see the ‘Methods’ section). Whole-brain statistical overlay results are displayed on a standard anatomical MNI template.

In contrast, individuals with higher ratings for security showed higher negative neural sensitivity to magnitude in the right amygdala spanning the right IFG *pars orbitalis* (Figure 4A, Supplementary Table S5). Also, higher ratings for security were associated with higher positive neural sensitivity to probability in interaction with higher stake magnitudes in right superior occipital gyrus (SOG) and lingual gyrus, and left precuneus (Figure 4B; Supplementary Table S5). As seen in the ROI response trends illustrated in Supplementary Figure S6B, individuals with higher security ratings engaged higher (more positive) to lower (more negative) neural sensitivities to winning probability for high to low stake magnitudes, respectively. That is, BOLD responses in these ROIs for these participants were higher for winning than losing probability at higher than lower magnitudes. In contrast, individuals with lower security ratings engaged lower to higher neural sensitivities to win probability from high to low magnitudes, respectively.

### Hedonism and security modulate LCT brain functional degree centrality

**Fig. 5.**
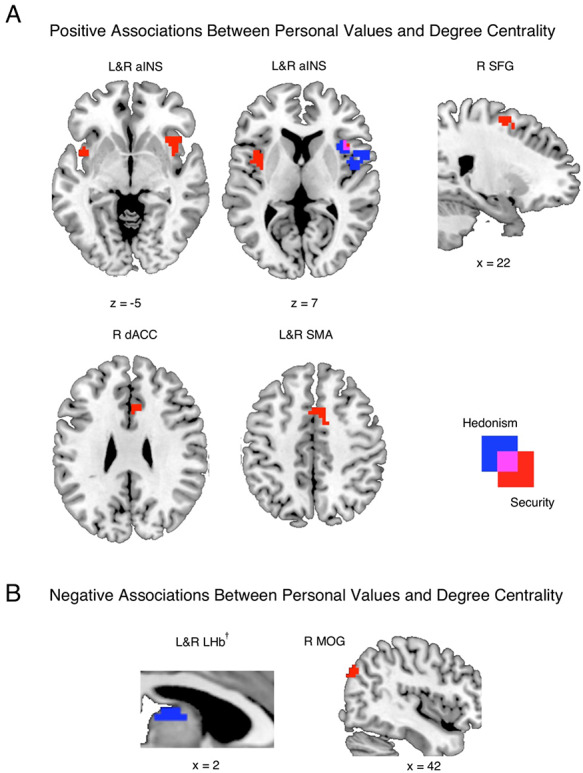
Whole-brain areas with (A) positive and (B) negative associations between personal values and neural degree centrality during the Lottery Choice Task (LCT). Regions with significant degree centrality associations with hedonism, security and their overlap are depicted in blue, red and magenta, respectively (see Supplementary Table S6). As shown by the figures, higher hedonism rating was associated with higher functional connections between whole-brain and right insula, but lower connections with habenula. On the other hand, higher security rating was associated with higher functional connections between whole-brain and bilateral aINS, SMA, and right SFG, dACC, but lower connections with right MOG. L = left; R = right; aINS = anterior insula; SMA = supplementary motor area; dACC = dorsal anterior cingulate cortex; SFG = superior frontal gyrus; LHb = lateral habenula; MOG = middle occipital gyrus. Contrast threshold significance level was set at cluster-wise *P*(FWE) < 0.05 (see the ‘Methods’ section). Whole-brain results are displayed on a standard anatomical MNI template. †: The results in the subcortical mask were derived from small volume correction with a primary voxel threshold of *P* < 0.005 (uncorrected) (see Supplementary Methods). Note that if the primary voxel threshold was set to *P* < 0.001 (uncorrected), the cluster threshold size would be *k* = 20, while the cluster size of the bilateral LHb would shrink to *k* = 59 and remain significant.

Significant associations were also found between hedonism and security and functional degree centrality in the brain during LCT performance (Figure 5, Supplementary Table S6). Higher hedonism ratings were associated with higher degree centrality in right insula (Figure 5A) but lower degree centrality in bilateral lateral habenula (LHb) during LCT performance (Figure 5B). Thus, in participants with higher hedonism ratings, task-related functional neural activity across the whole brain had higher correlations with activity in the right insula and lower correlations with the LHb, relative to participants with lower hedonism ratings. In contrast, higher security ratings were associated with higher degree centrality in bilateral insula and supplementary motor area (SMA), right dorsal anterior cingulate cortex (dACC) and superior frontal gyrus (SFG) (Figure 5A) but lower degree centrality in right middle occipital gyrus (MOG). Thus, in participants with higher security ratings, functional neural activity across the whole brain had higher correlations with activity in bilateral insula and SMA, and right dACC and SFG, and lower correlations with MOG, relative to participants with lower security ratings. Interestingly, both personal value ratings were associated with higher degree centrality of right insula although the overlapping cluster using minimal *t*-statistic conjunction analysis comprised only 11 voxels and did not surpass threshold (Figure 5A).

## Discussion

The present study presents data on distinct neural responses in the brain underlying differential value-based decision behaviors that were associated with personal values. Compared to their counterparts, individuals with higher hedonism ratings accepted more losing stakes and had higher neural responses to losing than winning probability at higher magnitudes across right dorsolateral prefrontal and striatal, and left parietal areas. Individuals with higher security ratings showed reduced neural responses at higher magnitudes in right inferior frontal and amygdala areas and enhanced neural responses to winning stakes as magnitudes increased in left precuneus. Moreover, higher security ratings corresponded with greater functional connectivity with the whole brain across bilateral insula and supplementary motor areas, and right dorsal anterior cingulate cortex and SFG compared to mainly the right insula for those with higher hedonism ratings.

We note that previous studies have reported associations between personal values with frontal and striatal processing of monetary decisions (Brosch *et al.*, 2011; Kuss *et al.*, 2015; Sul *et al.*, 2015). However, these studies focused on social values and decisions to apportion money to others. Our findings in frontal and striatal areas using the LCT protocol demonstrate that personal values to do with the self also modulate neural computation of anticipated reward for the self that is encoded in stimuli more fundamentally apart from any social referents. Indeed, processing in these brain areas is engaged in putative assessments of reward without social implications (Knutson *et al.*, 2001 2003 2005; Camerer, 2008; Rangel *et al.*, 2008; Haber and Knutson, 2010; Dolan and Dayan, 2013). These findings together suggest that a common cognitive mechanism involving frontal and striatal processing underlies how different endogenous value bases including economic, social and personal ideological priorities modulate neural computations leading to final behavioral action.

The association of higher hedonism with higher acceptance behavior and right dorsolateral prefrontal activity for high magnitude stakes with losing probability in this young adult sample extends our previous findings in older adults (Goh *et al.*, 2016; Su *et al.*, 2018). Specifically, older adults accepted more losing stakes than younger adults accompanied with higher lateral and dorsomedial frontal activity, with greater expression of this neural pattern in older individuals with more extreme acceptances of such stakes. Lateral and dorsomedial frontal processing has been observed in tasks requiring cognitive control to regulate affect, particularly for negative emotions (Dodds *et al.*, 2011; Shenhav *et al.*, 2013). We speculate the right DLPFC activity in our study reflects greater control processing when individuals with higher hedonism are deciding about losing high-magnitude stakes. This might be because the prospect of potential reward despite losing gambles involves greater conflict in more hedonistic individuals. Cognitive control might play a more critical role in hedonistic individuals to select appropriate actions relative to individuals for which the prospective reward is less of a priority in such cases. Further studies manipulating conflict and reward are needed to specify the role of the right DLPFC when individuals accept tempting, albeit losing, stakes.

In contrast, security was associated with a distinct network of neural responses to LCT value parameters. Higher security corresponded with greater *decrement* in right inferior frontal and amygdala activity with increasing stake magnitudes. These brain areas are implicated in more upstream processing of affective reactions (Jones *et al.*, 2011) relative to the downstream control of goal-directed behavioral actions involving dorsolateral prefrontal areas. Individual with higher security also engaged higher neural responses for losing than winning low-magnitude stakes in left precuneus and right superior occipital and lingual areas. These latter brain areas have been implicated in perceptual attention and integration (Rothkirch *et al.*, 2014). We note that our stimuli are relatively simplistic numerical text that likely do not require much complex visual parsing or integration. Nevertheless, these associations might indicate greater or sustained perceptual processing to maintain neural representations of the numerical stimuli before a decision is reached. We suggest that future studies comparing different stimuli formats or task computations are required to evaluate the role of these perceptual regions in processing text stimuli during value-based information processing. Overall, while speculative, we interpret our findings to suggest ‘enhanced perceptual processing’ of stimuli with minute risks (possible loss of small magnitudes) amidst ‘lower general affective reaction’ to risky high-magnitude stakes in individuals with high security.

Higher hedonism and security were both associated with higher functional degree centrality during the LCT in the right anterior insula, implicated in affective reactions (Preuschoff *et al.*, 2006 2008; Mohr *et al.*, 2010; Jones *et al.*, 2011). This suggests stronger prioritizing of these personal values might commonly operate via enhanced affective modulation of cognitive processing of value-based stimuli throughout the brain. Beyond the right insula, however, higher security additionally correlated with greater functional degree centrality in the left insula, right SFG, dorsal anterior cingulate and bilateral supplementary motor areas. Previous studies have linked higher and more efficient global functional connectedness to better cognitive ability (van den Heuvel *et al.*, 2009; Cole *et al.*, 2012), implying more effective communication between different processes across various brain areas. Thus, it is possible that more extensive functional degree centrality in those prioritizing security may reflect greater negotiation between different cognitive processes engaged across broader brain networks during value-based decision-making. We also note that greater hedonism negatively correlated with negative degree centrality in the lateral habenula, which is known to signal the absence of reward or presence of punishment (Matsumoto and Hikosaka, 2007 2009; Proulx *et al.*, 2014) with dysfunction implicated in impulsivity (Zapata *et al.*, 2017). Our finding of lower degree centrality of the lateral habenula in more hedonistic individuals suggests that there might be reduced functional communication of this brain area with the rest of the cerebrum in these individuals. Reduced lateral habenula communication efficacy with the cerebrum with greater hedonism is consistent with lower signaling of negative outcomes, accounting for higher acceptance of losing stakes as well.

It is important to emphasize that our findings reflect associations between SVS-assessed personal values and LCT behavior and neural processing at best. Studies utilizing more implicit measures of personal values or manipulations thereof (e.g. priming) are still required to evaluate a more causal effect on value-based decision processing. Nevertheless, we highlight that participants in our study were agnostic to the relationship between the SVS assessment and the LCT, which were acquired on separate days under different experimental contexts. Thus, we considered that the SVS captures personal values over a generic timeframe with respect to the participants’ worldview. As such, we suggest that while our findings are associative, our measures of hedonism and security do reflect some prior endogenous motivations that exerted influence on transient LCT performance and neural processing assessed in our study.

In sum, the degree to which an individual prioritizes hedonism or security modulates their neural responses to economic-related value stimuli information, which is also associated with differences in risky decision behavior. The brain areas showing such neural response modulation by personal values have been implicated in processing of reward anticipation in other studies and span affective and control systems in the brain as well. Differential interplay of these neurocognitive computations across individuals with these different personal values likely alter objective value coded in stimuli. These initial findings provide impetus for more specific studies on how specific personal values might be acquired over life experiences and encoded in the brain. Such investigations might afford more precise explanations and predictions about human decision behaviors given the prior state of an individual’s neuropsychological profile.

## Supplementary Material

nsaa150_Supp
