# Supplementary material for: Neural responses reveal associations between personal values and value-based decisions
Source: Soc Cogn Affect Neurosci. 2020 Nov 4;15(11):1217–27. doi: 10.1093/scan/nsaa150 (PMC7745144; doi:10.1093/scan/nsaa150)
Supplement: nsaa150_Supp [file nsaa150_supp.zip › scan-19-296-File002.docx]

Supplementary Methods

Sample Size Considerations

We regarded our sample size of 40 young adults to be adequate for detecting associations between decision-making neural functional responses and SVS measures for the following reasons. First, the brain responses relevant to our LCT experiment had already been observed in our previous work using this sample of 40 young adults in Su et al. (2018). Second, we also evaluated the sample and statistical sizes used in three previous relevant studies that also evaluated associations between personal values and value-based decision processing neural responses. Brosch, Coppin, Scherer, Schwartz, and Sander (2011) included a sample size of 19, with whole-brain analysis criteria of voxel-wise *p(uncorrected)* < 0.001, cluster size 5, or whole-brain *p(FWE)* < .05, and found significant whole-brain decision-making responses associated with SVS scores with peak voxel z-scores that ranged between 2.99 to 3.47. Sul et al. (2015) had 26 participants, with voxel-wise *p(uncorrected)* < 0.001 and whole-brain cluster-wise *p(FWE)* < .05, and found significant whole-brain decision-making responses associated with personal strategic differences in experimental behavior with peak voxel z-scores that ranged between 3.73 to 4.06. Finally, Kuss et al. (2015) included a sample size of 36, with voxel-wise *p(uncorrected)* < .005 and whole-brain cluster-wise *p(FWE)* < .05, and found significant whole-brain decision-making responses associated with Social Value Orientation (SVO) scores with *t*-values of effects in peak voxels that ranged from 3.86 to 5.01. In our study, we use a voxel level alpha of *p* < 0.001 in our whole-brain statistical thresholding criteria approach. Applying our criteria with the relevant statistics from Kuss et al. (2015) which had the largest sample size, our sample size of 40 yields a power of 0.610 to 0.909 (calculated using G*Power; Faul, Erdfelder, Lang, & Buchner, 2007) to detect relevant effects of personal values on neural value-based decision processing responses in voxels given our whole-brain statistical thresholding criteria. Third, our study has the largest sample size in comparison to all these previous related studies. We report our power for reference in future studies and regard our approach and data sample size as within the recommendations forwarded in (Button et al., 2013; Geuter, Qi, Welsh, Wager, & Lindquist, 2018).

**Schwartz Value Scale Sub-Values for Hedonism and Security**

The SVS is composed of 57-word items that each describe different sub-values of the ten personal values: self-direction, stimulation, hedonism, achievement, power, security, conformity, universalism, tradition, and benevolence. Each word item is followed by annotation in parentheses (e.g., "Self-Indulgent (doing pleasant things)"). Relevant to this study, hedonism was defined as “Pleasure and sensuous gratification for oneself”. Each participant’s hedonism score was the average of their ratings for SVS sub-values “Pleasure (gratification of desires)”, “Enjoying life (enjoying food, sex, leisure, etc.)”, and “Self-Indulgent (doing pleasant things)”. Security was defined as “Safety, harmony and stability of society, of relationships, and of self” and was computed based on the average of the ratings for “National security (protection of my nation from enemies)”, “Reciprocation of favors (avoidance of indebtedness)”, “Family security (safety for loved ones)”, “Clean (neat, tidy)”, and “Social order (stability of society)”. We highlight that SVS scores were acquired for all other constructs. In line with our expectation that value-based decision processing should involve mainly values to do with gaining rewards (i.e., hedonism) and maintaining status quo (i.e., security), analysis using the other SVS scores did not yield meaningful results.

Behavioral Response Time Data Analysis

Response times (RT) analysis used a similar approach as Equation 1 with three exceptions. First, the dependent variable was the log-transformed trial-wise RTs of all participants. Second, trial-wise binary decision responses (*D*) was included as an independent variable. Third, coefficients for the square of probability *(b_P_^2^*) and its interaction with decision response (*b_DP_^2^*) were included to model longer RTs at middle probabilities, which are less certain than extreme probabilities. The full model for RT data is as follows.

| $log\left( RT \right)=b_{0}+b_{Sex}Sex+b_{H}H+b_{S}S+b_{P}P+b_{M}M+b_{D}D+b_{P^{2}}P^{2}+b_{PM}PM+b_{PD}PD+b_{MD}MD+b_{{MP}^{2}}MP^{2}+b_{{DP}^{2}}DP^{2}+b_{HP}HP+b_{SP}SP+b_{HM}HM+b_{SM}SM+b_{HD}HD+b_{SD}SD+b_{{HP}^{2}}HP^{2}+b_{{SP}^{2}}SP^{2}+b_{PMD}PMD+b_{HPM}HPM+b_{SPM}SPM+b_{HPD}HPD+b_{SPD}SPD+b_{HMD}HMD+b_{SMD}SMD+b_{{HDP}^{2}}HDP^{2}+b_{{SDP}^{2}}SDP^{2}+b_{HPMD}HPMD+b_{SPMD}SPMD+v_{0i}+v_{Pi}P+v_{Mi}M+v_{Di}D+v_{PMi}PM+v_{PDi}PD+v_{MDi}MD+v_{PMDi}PMD+\varepsilon_{i}$ | (2) |
| --- | --- |

In Equation 2, the fixed effect coefficients associated with hedonism *(b_H_, b_HP_, b_HM_, b_HD_, b_HPM_, b_HPD_, b_HMD_, b_HP_^2^, b_HDP_^2^*) and security *(b_S_, b_SP_, b_SM_, b_SD_, b_SPM_ , b_SPD_, b_SMD_, b_SP_^2^, b_SDP_^2^*) characterize the associations between personal values and log(RT) during accepted and rejected trials over trial-wise variations in probability and magnitude. Positive and negative coefficients for linear effects indicate that the personal values were associated with increased and decreased log(RT), respectively, with increasing probability and magnitude given the base log(RT) during rejected (*b_0_*) and accepted trials (*b_D_*) and effects of probability and magnitude *(b_P_, b_M_, b_PM_, b_PD_, b_MD_, b_PMD_*). Positive and negative quadratic coefficients indicate U- and inverted U-shaped modulation of probability effects, respectively. *ε* are the residuals.

Brain Imaging Protocol, Preprocessing, First-Level Parametric Modeling

Functional gradient-echo echo planar imaging (EPI) sequence images included 38 axial slices parallel to the AC-PC plane with voxel-size 3.4375 × 3.4375 × 4 mm; no inter-slice gap; in-plane matrix size 64 × 64; field of view (FOV) 20.63 × 20.63 cm^2^; repetition time (TR) 2000 ms; echo time (TE) 24 ms, and flip angle 90°. T2 images included 38 axial slices, voxel-size 1 × 1 × 4 mm; no inter-slice gap; in-plane matrix size 256 × 256; FOV 25.6 × 25.6 cm^2^; TR 7480 ms; TE 102 ms; flip angle 150°. T1 images included 192 sagittal slices, voxel-size 1 × 1 × 1 mm; no inter-slice gap; in-plane matrix size 256 × 256; FOV 25.6 × 25.6 cm^2^; TR 2000 ms, TE 2.98 ms, flip angle 9°.

For each participant, functional images were first corrected for slice-timing (registered to the first slice) and head motion. Functional images were then co-registered to the T2 and then to the T1 anatomical image. Co-registered T1 images were then spatially normalized to the standard Montreal Neurological Institute (MNI) template space. The resulting transformation parameters were then applied to the functional images, which were then resampled to 3 mm isotropic voxel-sizes and spatially smoothed with a 3D 8 mm full width at half maximum Gaussian kernel.

In first-level model-based fMRI GLM analyses, parametric modulation of convolved HRF functions were applied to choice-phase regressors and non-null outcome-phase regressors. Parametric modulators were not orthogonalized to avoid sequential remainder variance effects that are dependent on regressor order in SPM. This also results in the unmodulated choice-phase regressor, which is not of interest, as modeling the mean neural response when parametric variables were 0 (Mumford, Poline, & Poldrack, 2015).

Whole-brain Contrast Statistical Significance Thresholds for fMRI LCT Neural Responses

Whole-brain second-level GLM statistical images of each LCT variable had different levels of effective spatial smoothness which were first estimated using the spatial autocorrelation function from the program 3dFWHMx in AFNI based on the respective residual images. In addition, we had the *a priori* assumption that neural responses should take place predominantly in cerebral gray matter so that we applied gray matter masks for all whole-brain voxel-wise analysis. Gray matter masks were defined using the Harvard-Oxford Atlas (HarvardOxford-sub-maxprob-thr50-1mm.nii distributed with FSL 5.0; Jenkinson, Beckmann, Behrens, Woolrich, & Smith, 2012). For cortical gray matter, frontal, parietal, temporal, and occipital lobes were included in the mask. This mask was applied along with the corresponding autocorrelation parameter estimates, a primary voxel threshold *p* < .001 (uncorrected), and second-nearest neighbor clustering setting, into 3dClustSim which then computed Monte Carlo simulations with 10,000 iterations. Studies have demonstrated that this approach provides reasonable false positive rate adjustment for multiple comparisons issues (Cox, Chen, Glen, Reynolds, & Taylor, 2017). Accordingly, 3dClustSim yielded cluster size thresholds, *k*, which were 28, 33, and 30 for PROB, MAG, and P×M, respectively, for whole-brain cluster-level *p(FWE)* < .05 within the cortical gray matter masked area. For subcortical and midbrain regions, our previous work using the LCT (Goh et al., 2016) suggested these areas evince generally smaller extents of responses. Moreover, because studies have suggested that subcortical regions tend to suffer from lower signal-to-noise ratio due to physiological noise such as CSF flow (Maugeri et al., 2018; Noble, Scheinost, & Constable, 2020), we expected the voxel intensity (voxel-wise t value) to be lower in subcortical regions. As such, we adopted an *a priori* small volume correction approach and defined one single subcortical mask that includes the striatum, thalamus, hippocampus, amygdala, and nucleus accumbens using the same atlas and applied a primary voxel threshold of *p* < 0.005 (uncorrected) in this mask, which was the voxel threshold used for these areas in (Goh et al., 2016). This mask and corresponding settings applied to 3dClustSim yielded cluster size thresholds of 11, 10, and 10 to reach cluster-level *p(FWE)* < .05 within subcortical areas, for PROB, MAG, and P×M contrasts, respectively. To address the concerns about using this more relaxed primary voxel threshold of *p* < 0.005 (uncorrected), we also reported the results for the subcortical regions with a primary voxel threshold *p* < .001 (uncorrected). To reach cluster-level *p(FWE)* < .05 at voxel threshold *p* < .001, 3dClustSim yielded cluster size thresholds of 5, 5, and 5.

Preprocessing and Analysis for fMRI LCT Degree Centrality

Whole-brain voxel-wise task-related degree centrality images during the LCT were computed for each participant as follows. For each participant, the spatially normalized but unsmoothed functional images from the preprocessing procedure were first masked to retain signals exclusively from cerebral grey matter. To generate a grey matter mask, we excluded cerebral white matter, lateral ventricles and brain-stem defined using the Harvard-Oxford Atlas (HarvardOxford-sub-maxprob-thr50-1mm.nii distributed with FSL 5.0; Jenkinson et al., 2012). The resulting masked functional images were then subjected to voxel-wise temporal linear trend removal and a temporal band-pass filter (0.009 Hz < f < 0.08 Hz) using RESTplus toolbox version 1.22. A GLM was then applied on the temporally filtered images that included the first-level GLM as defined in the Methods, additional regressors for the average signal time courses for the whole brain, the ventricles, cerebral white matter (also defined using the Harvard-Oxford Atlas), as well as the first order derivatives for the motion and averaged signal time courses. The residual images from this GLM were then used to generate an *n* × *n* Pearson's correlation coefficient matrix with *n* being the total number of voxels within the cerebral grey matter mask. Degree centrality images were then derived for each participant from the correlation matrix such that the value in each voxel of a participant’s degree centrality image was the number of other voxels it was positively correlated with based on the threshold of *r* > .25 as evaluated in previous studies assessing this measure (Buckner et al., 2009). Degree centrality images were then standardized using *z*-score transformation based on each individual's grey matter voxel-wise degree centrality distribution. The resulting *z*-statistic maps were then smoothed with a 3D 8 mm full width at half maximum Gaussian kernel. Whole-brain contrast statistical significance was set at cluster-wise *p(FWE)* < .05. Correspondingly, the cluster threshold at voxel-wise *p* < .001 (uncorrected) was *k* > 31 for cortical areas and the cluster threshold at voxel-wise *p* < .005 (uncorrected) was *k* > 77 for subcortical and midbrain areas. To address the concerns about using a more relaxed primary voxel threshold of *p* < 0.005 (uncorrected), we also reported the results for the subcortical regions with a primary voxel threshold *p* < .001 (uncorrected). To reach cluster-level *p(FWE)* < .05 at voxel threshold *p* < .001, 3dClustSim yielded a cluster size threshold of 20.

Supplementary Results

**Primary LCT Acceptance Rate Behavior**

Effects of probability and magnitude on AR based on Equation 1 were consistent with our previous analysis (Supplementary Table 2). Specifically, increasing winning probability (or decreasing losing probability) increased ARs (P: *b* = 5.90, *SE =* 0.50, *z* = 11.76, *p* < .001, 95% CI = [4.92, 6.89]), even after accounting for personal value effects. Also, there was a non-significant increase in AR with decreasing stake magnitude (M: *b* = −0.17, *SE =* 0.10, *z* = −1.66, *p* = .098, 95% CI = [−0.37, 0.03]) that was significantly accentuated as winning probabilities lowered (P × M: *b* = 0.68, *SE =* 0.24, *z* = 2.84, *p* = .005, 95% CI = [0.21, 1.15]).

**LCT Response Time Behavior**

Effects of probability and magnitude on RTs based on Equation 2 were also consistent with our previous study (Supplementary Table 3). Response times, averaged across accepted and rejected trials, were longer for high than low winning probability conditions (P: *b* = 9.17%, *SE* = 1.42%, *t*(24.37) = 6.46, *p* < .001, 95% CI = [6.38%, 11.95%]) but longest for middle probability conditions (P^2^: *b* = 1.50%, *SE* = 0.72%, *t*(27.04) = 2.10, *p* = .045, 95% CI = [0.10%, 2.91%]) (Supplementary Figure 2A). Further, high probability stakes took longer to reject than accept while low probability stakes took longer to accept than reject (P × D : *b* = −20.67%, *SE =* 2.05%, *t*(28.15) = −10.06, *p* < .001, 95% CI = [−24.69%, −16.64%]) (Supplementary Figure 2B). The main effect of magnitude and magnitude by decision interaction were not significant on RT.

Hedonism did not significantly modulate any probability or magnitudes effects on RTs using Equation 2 (Supplementary Table 3). Also using Equation 2, individuals with higher security ratings had faster RTs (S: *b* = −3.79%, *SE* = 1.30%, *t*(39.14) = −2.92, *p* = .006, 95% CI = [−6.33%, −1.25%]) particularly for rejections (S × D: *b* = 4.11%, *SE* = 1.18%, *t*(46.52) = 3.48, *p* = .001, 95% CI = [1.80%, 6.43%]; positive coefficient indicates slower acceptances) ( Supplementary Table 3; Supplementary Figure 3, 5).

| *Supplementary Table 1. The Expected Values of Each Choice Condition in the Lottery Choice Task.* | | | | | | |
| --- | --- | --- | --- | --- | --- | --- |
| Magnitude  Level |  | Winning Probability Level | | | | |
|  |  | Low-Low  (LL) | Middle-Low  (ML) | Middle-Middle  (MM) | Middle-High  (MH) | High-High  (HH) |
| High (H) |  | −86.5  [−97.2, −75.6] | −45  [−56.2, −32.3] | −1.1  [−12.3, 10.9] | 39.5  [28.3, 50.4] | 84.7  [84, 98.1] |
| Middle (M) |  | −45.3  [−52.8, −37.7] | −23.6  [−31.2, −16.0] | −2.2  [−7.0, 2.4] | 22.7  [15.1, 28.8] | 44.2  [36.7, 52.5] |
| Low (L) |  | −5.0  [−9.8, −0.7] | −2.0  [−5.0, −0.4] | 0.1  [−1.4, 0.7] | 2.7  [0.6, 5.5] | 5.1  [0.7, 9.8] |
| *Note:* The means and ranges (in brackets) of expected values of each choice condition in the Lottery Choice Task are shown above. The means and ranges of winning probability (%) across different levels are as follows: LL = 9.4 [4, 15], ML = 28.7 [24,34], MM = 49.3 [44, 55], MH = 69.1 [64, 75], HH = 89.9 [84, 95]. The means and ranges across magnitude (points) levels are: L = 6.2 [1, 12], M = 56.0 [50,61], H = 103.9 [99, 110]. Modified from Su et al. (2018). | | | | | | |

| *Supplementary Table 2. The Effects of Personal Values in Modulating Logit Binary Decision Responses During the Lottery Choice Task.* | | | | | |
| --- | --- | --- | --- | --- | --- |
| Term | *b* | [95% CI] | *SE* | *z* | *p* |
| Intercept | 0.32 | [−0.03, 0.67] | 0.18 | 1.82 | .069 |
| Sex | −0.01 | [−0.33, 0.30] | 0.16 | −0.08 | .940 |
| H | 0.03 | [−0.31, 0.37] | 0.18 | 0.17 | .867 |
| S | −0.38 | [−0.73, −0.03] | 0.18 | −2.14 | .032 |
| P | 5.90 | [4.92, 6.89] | 0.50 | 11.76 | <.001 |
| M | −0.17 | [−0.37, 0.03] | 0.10 | −1.66 | .098 |
| P × M | 0.68 | [0.21, 1.15] | 0.24 | 2.84 | .005 |
| H × P | 0.15 | [−0.74, 1.04] | 0.45 | 0.33 | .740 |
| S × P | 0.91 | [−0.01, 1.82] | 0.47 | 1.94 | .053 |
| H × M | −0.10 | [−0.30, 0.10] | 0.10 | −1.00 | .316 |
| S × M | 0.12 | [−0.08, 0.32] | 0.10 | 1.16 | .247 |
| H × P × M | 0.35 | [0.02, 0.68] | 0.17 | 2.06 | .039 |
| S × P × M | 0.02 | [−0.33, 0.37] | 0.18 | 0.11 | .915 |
| *Note:* See Methods and Equation 1 for the details of the linear mixed model. H = hedonism; S = security; P = probability; M = magnitude. Missed decisions (mean (SD) = 2.37 (3.34) per person) were not included in this model. | | | | | |

| *Supplementary Table 3. The Effects of Personal Values in Modulating (log−transformed) Decision Response Times During the Lottery Choice Task.* | | | | | | |
| --- | --- | --- | --- | --- | --- | --- |
| Term | *b* (%) | [95% CI] | *SE* (%) | *df* | *t* | *p* |
| Intercept | 321.54 | [318.96, 324.12] | 1.32 | 40.20 | 243.99 | <.001 |
| Sex | −0.86 | [−2.62, 0.90] | 0.90 | 39.61 | −0.96 | 0.345 |
| H | 0.36 | [−2.32, 3.04] | 1.37 | 47.76 | 0.26 | 0.795 |
| S | −3.79 | [−6.33, −1.25] | 1.30 | 39.14 | −2.92 | 0.006 |
| P | 9.17 | [6.38, 11.95] | 1.42 | 24.37 | 6.46 | <.001 |
| M | 0.61 | [−0.62, 1.84] | 0.63 | 48.23 | 0.97 | 0.335 |
| D | −2.14 | [−4.54, 0.26] | 1.22 | 49.94 | −1.75 | 0.086 |
| P^2^ | 1.50 | [0.10, 2.91] | 0.72 | 27.04 | 2.10 | 0.045 |
| P × M | 0.74 | [−0.56, 2.03] | 0.66 | 46.24 | 1.11 | 0.271 |
| P × D | −20.67 | [−24.69, −16.64] | 2.05 | 28.15 | −10.06 | <.001 |
| M × D | −0.01 | [−1.43, 1.41] | 0.72 | 34.58 | −0.01 | 0.989 |
| M × P^2^ | 0.34 | [−0.19, 0.87] | 0.27 | 35.09 | 1.26 | 0.217 |
| D × P^2^ | −0.95 | [−2.46, 0.56] | 0.77 | 39.97 | −1.23 | 0.225 |
| H × P | −0.29 | [−3.19, 2.62] | 1.48 | 28.58 | −0.19 | 0.848 |
| S × P | −0.58 | [−3.31, 2.15] | 1.39 | 22.72 | −0.42 | 0.68 |
| H × M | −0.53 | [−1.46, 0.40] | 0.47 | 41.24 | −1.11 | 0.274 |
| S × M | 0.09 | [−0.83, 1.00] | 0.47 | 37.49 | 0.19 | 0.854 |
| H × D | −0.11 | [−2.61, 2.40] | 1.28 | 65.07 | −0.08 | 0.934 |
| S × D | 4.11 | [1.80, 6.43] | 1.18 | 46.52 | 3.48 | 0.001 |
| H × P^2^ | −0.13 | [−1.59, 1.33] | 0.75 | 31.53 | −0.17 | 0.864 |
| S × P^2^ | −0.40 | [−1.77, 0.98] | 0.70 | 25.4 | −0.57 | 0.575 |
| P × M × D | −1.52 | [−3.69, 0.65] | 1.11 | 22.79 | −1.37 | 0.184 |
| H × P × M | −0.13 | [−0.98, 0.72] | 0.44 | 66.35 | −0.30 | 0.766 |
| S × P × M | −0.22 | [−1.07, 0.62] | 0.43 | 60.73 | −0.52 | 0.606 |
| H × P × D | −0.67 | [−4.67, 3.33] | 2.04 | 27.82 | −0.33 | 0.744 |
| S × P × D | −0.85 | [−4.81, 3.11] | 2.02 | 26.2 | −0.42 | 0.676 |
| H × M × D | 0.59 | [−0.83, 2.00] | 0.72 | 34.58 | 0.81 | 0.424 |
| S × M × D | −0.19 | [−1.61, 1.22] | 0.72 | 33.75 | −0.27 | 0.791 |
| H × D × P^2^ | 0.53 | [−1.05, 2.11] | 0.81 | 47.42 | 0.66 | 0.513 |
| *Supplementary Table 3 (cont’d)* | | | | | | |
| S × D × P^2^ | 0.90 | [−0.57, 2.36] | 0.75 | 36.38 | 1.20 | 0.238 |
| H × P × M × D | 0.02 | [−1.09, 1.13] | 0.57 | 57.72 | 0.03 | 0.974 |
| S × P × M × D | 0.57 | [−0.54, 1.68] | 0.57 | 59.66 | 1.01 | 0.317 |
| *Note:* All coefficients, 95% CI, and *SE,* are reported in percentage (%) to increase the readability of minuscule numbers. See Methods and Equation 2 for the details of the linear mixed model. Also note that to interpret the coefficients of regression in its native unit (ms), one should convert it back from log-transformed unit and compare it with the baseline, which is 10^Intercept^ = 10^321.54%^ = 1642.10ms. For example, for probability, *b* = 9.17%, this should be interpreted as “one standardized unit increase in probability multiplies the baseline RT by a factor of 10^9.17%^”, i.e., RT goes from 1659.59ms to 1642.10 × 10^9.17%^ = 2028.15ms. H: hedonism; S: security; P: probability, M = magnitude; D = decision response; P^2^ = the square of probability. Missed decisions (mean (SD) = 2.37 (3.34) per person) were not included in this model. | | | | | | |

| *Supplementary Table 4. Clusters in Which Hedonism Modulated Neural Sensitivity to LCT Value Parameters.* | | | | | | | |
| --- | --- | --- | --- | --- | --- | --- | --- |
| Region | Hemis-phere | Peak MNI coordinate | | |  | Cluster  Size (*k*) | Peak  t value |
|  |  | x | y | z |  |  |  |
| Negative Effect on  P × M Sensitivity |  |  |  |  |  |  |  |
| DLPFC | R | 54 | 32 | 23 |  | 35 | 4.96 |
| Angular gyrus | L | −42 | −64 | 47 |  | 31 | 4.09 |
| IPL | L | −45 | −52 | 41 |  |  | 4.03 |
| Caudate**^†^** | R | 18 | 20 | 8 |  | 16 | 3.68 |
| Lingual gyrus | R | 15 | −88 | −16 |  | 109 | 4.53 |
| Lingual gyrus | R | 9 | −85 | −7 |  |  | 4.12 |
| Cuneus | R | 12 | −94 | 11 |  |  | 4.08 |
| Lingual gyrus | R | 33 | −79 | −16 |  | 40 | 4.48 |
| IOG | R | 48 | −73 | −13 |  |  | 3.39 |
| *Note:* Clusters reported fulfilled whole-brain effect contrasts with cluster-wise adjustment of family-wise error (FWE) at *p(FWE)* < .05 (see Methods). MNI = Montreal Neurological Institute; L = left; R = right; P × M = probability × magnitude; DLPFC = dorsolateral prefrontal cortex; IPL = inferior parietal lobule; IOG = inferior occipital gyrus. A blank in the cluster column indicate that the region is the sub-peak within the cluster above. Regions are labeled using the Automated Anatomical Labeling atlas except for DLPFC, which we used here in keeping with the neuroimaging literature on cognitive control. **^†^**: The results in the subcortical mask were derived from small volume correction with a primary voxel threshold of *p* < .005 (uncorrected) (see Supplementary Methods). Note that if the primary voxel threshold was set to *p* < .001 (uncorrected), the cluster threshold size would be *k* = 5, while the cluster size of caudate would shrink to *k* = 2. | | | | | | | |

| *Supplementary Table 5. Clusters in Which Security Modulated Neural Sensitivity to LCT Value Parameters.* | | | | | | | |
| --- | --- | --- | --- | --- | --- | --- | --- |
| Region | Hemis-phere | Peak MNI coordinate | | |  | Cluster  Size (*k*) | Peak  *t* value |
|  |  | x | y | z |  |  |  |
| Negative Effect on  M Sensitivity |  |  |  |  |  |  |  |
| Amygdala | R | 27 | 5 | −13 |  | 62 | 4.41 |
| IFG, pars orbitalis | R | 36 | 26 | −16 |  |  | 4.20 |
| IFG, pars orbitalis | R | 30 | 17 | −22 |  |  | 3.83 |
| Positive Effect on  P × M Sensitivity |  |  |  |  |  |  |  |
| SOG | R | 21 | −82 | 41 |  | 149 | 5.03 |
| Lingual gyrus | R | 9 | −70 | 2 |  | 100 | 4.44 |
| Precuneus | L | −12 | −40 | 77 |  | 39 | 4.14 |
| *Note:* Clusters reported fulfilled whole-brain effect contrasts with cluster-wise adjustment of family-wise error (FWE) at *p(FWE)* < .05 (see Methods). P = probability; M = magnitude; P × M = probability × magnitude; MNI = Montreal Neurological Institute; L = left; R = right; IFG = inferior frontal gyrus; SOG = superior occipital gyrus. Blanks in the cluster columns indicate region is the sub-peak within the cluster above. Regions are labeled using the Automated Anatomical Labeling atlas. | | | | | | | |

| *Supplementary Table 6. Clusters in Which Personal Values Significantly Modulated Neural Degree Centrality During the LCT.* | | | | | | | |
| --- | --- | --- | --- | --- | --- | --- | --- |
| Region | Hemis-phere | Peak MNI coordinate | | |  | Cluster  Size (*k*) | Peak  *t* value |
|  |  | x | y | z |  |  |  |
| Positive Hedonism Effect |  |  |  |  |  |  |  |
| Anterior insula | R | 36 | 17 | 8 |  | 38 | 4.21 |
| Rolandic operculum | R | 45 | −4 | 14 |  | 78 | 3.98 |
| Negative Hedonism Effect |  |  |  |  |  |  |  |
| LHb**^†^** | L, R | 3 | −10 | 14 |  | 132 | 4.49 |
| Positive Security Effect |  |  |  |  |  |  |  |
| Anterior insula | L | −45 | 8 | 2 |  | 82 | 5.06 |
| dACC | R | 6 | 20 | 29 |  | 106 | 4.37 |
| SMA | L | −6 | 14 | 59 |  |  | 4.27 |
| SMA | R | 9 | 11 | 47 |  |  | 4.26 |
| Anterior insula | R | 42 | 17 | −4 |  | 78 | 4.16 |
| SFG | R | 21 | 2 | 56 |  | 33 | 4.10 |
| Negative Security Effect |  |  |  |  |  |  |  |
| MOG | R | 42 | −79 | 35 |  | 45 | 4.70 |
| *Note:* Clusters reported fulfilled whole-brain effect contrasts with cluster-wise adjustment of family-wise error (FWE) at *p(FWE)* < .05 (see Methods). MNI = Montreal Neurological Institute; L = left; R = right; LHb = lateral habeluna; dACC = dorsal anterior cingulate cortex; SMA = supplementary motor area; SFG = superior frontal gyrus, MOG = middle occipital gyrus. Blanks in the cluster columns indicate region is the sub-peak within the cluster above. Regions are labeled using the Automated Anatomical Labeling atlas except for LHb, which we used here in keeping with the neuroimaging literature on brain stem nuclei. **^†^**: The results in the subcortical mask were derived from small volume correction with a primary voxel threshold of *p* < .005 (uncorrected) (see Supplementary Methods). Note that if the primary voxel threshold was set to *p* < .001 (uncorrected), the cluster threshold size would be *k* = 20, while the cluster size of the bilateral LHb would shrink to *k* = 59. | | | | | | | |


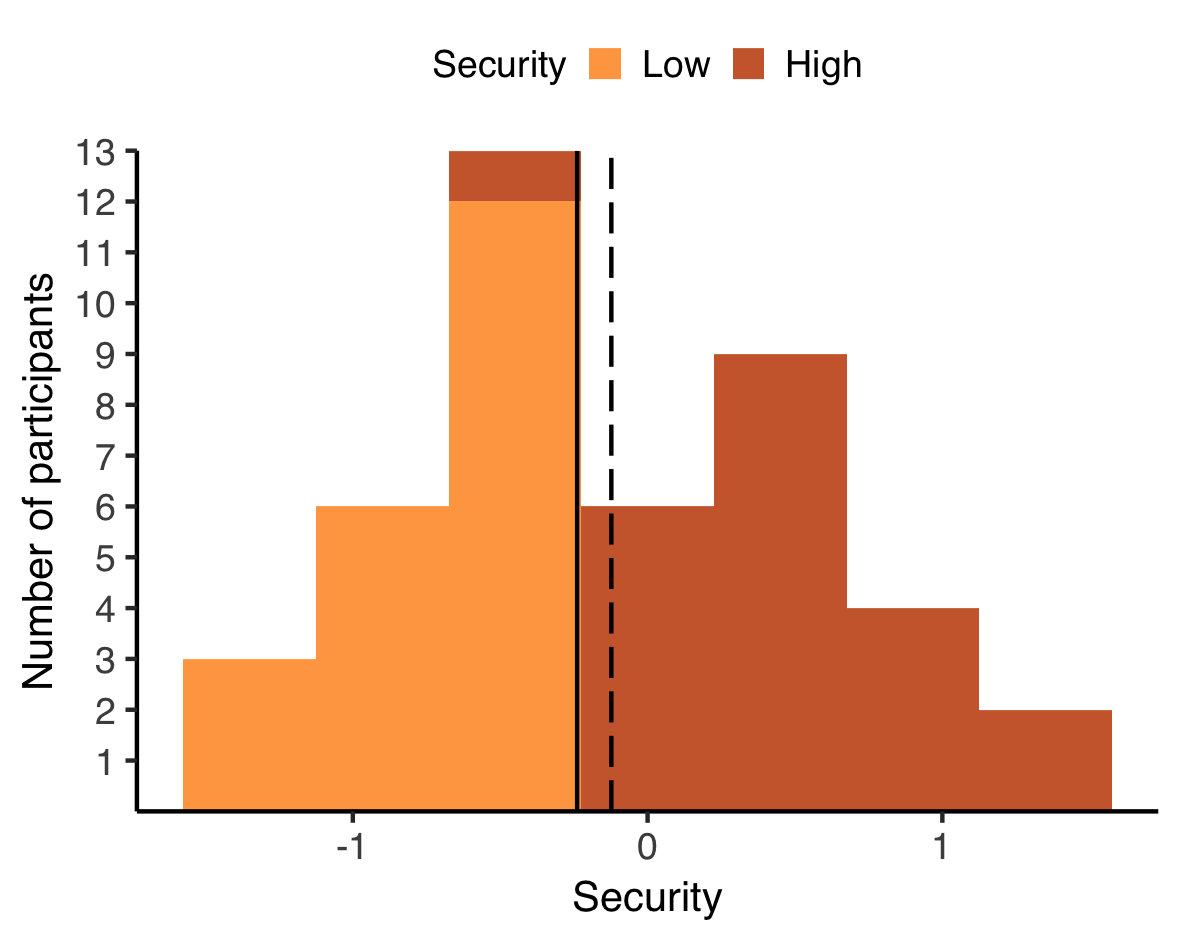

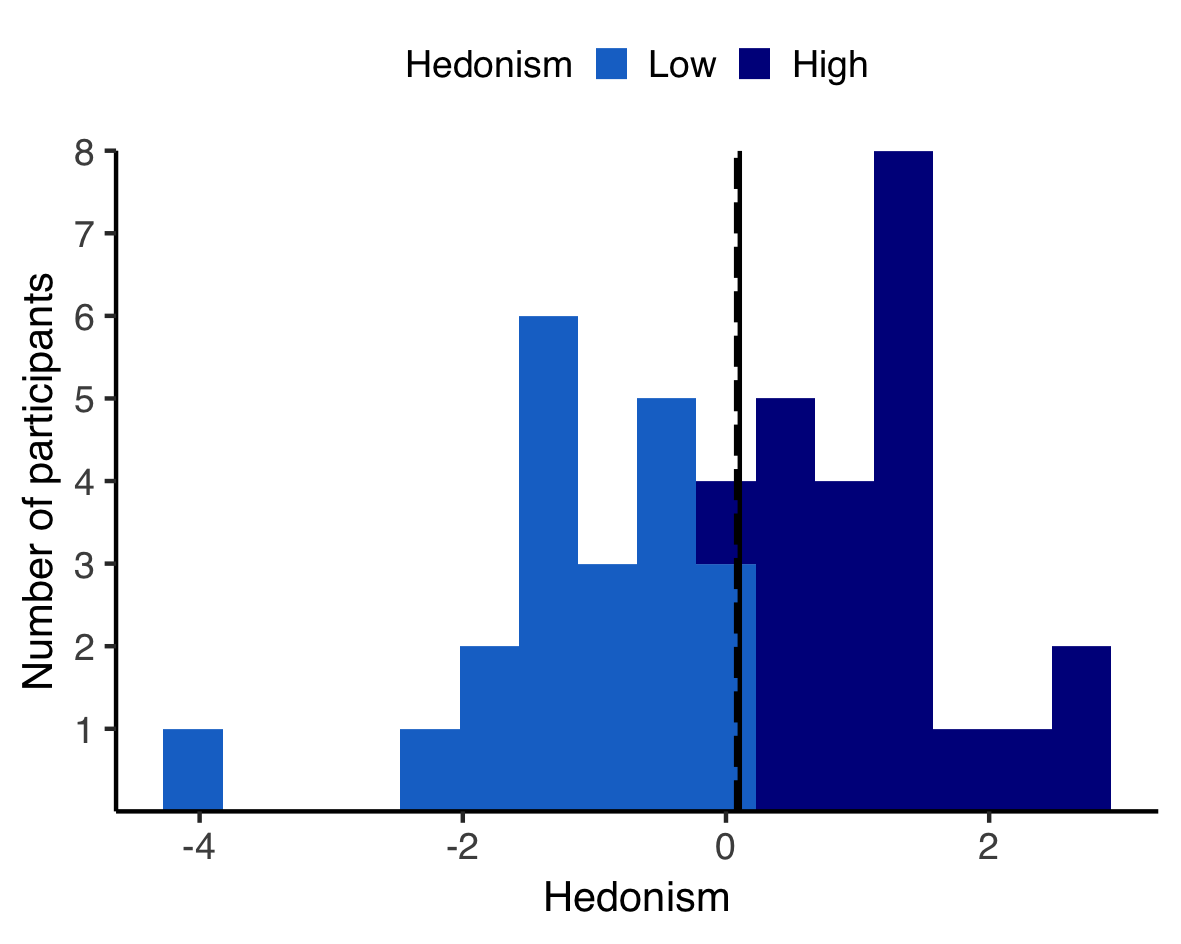


A

B

*Supplementary Figure 1.* Histograms showing participant centered-rating distributions for (A) hedonism and (B) security. Means and medians for the whole group are shown as dashed and solid lines, respectively. Darker and lighter colors depict high and low rating groups, respectively, split based on the median. Note that median-split groups are only used for visualization purposes (see Figure 2 and Supplementary Figure 3, 4, 5) but formal statistical analysis treated these rating variables continuously. The descriptive statistics for each group are as follows: hedonism low rating group: *n* = 21, *M* = −1.07, *Mdn* = −0.97, *SD* = 0.89, range = [−3.95, −0.07]; hedonism high rating group: *n* = 22, *M* = 1.17, *Mdn* = 1.20, *SD* = 0.73, range = [0.11, 2.90]; security low rating group: *n* = 21, *M* = −0.69, *Mdn* = −0.64, *SD* = 0.34, range = [−1.35, −0.24]; security high rating group: *n* = 22, *M* = 0.41, *Mdn* = 0.43, *SD* = 0.45, range = [−0.24, 1.28]. Hedonism and security ratings were not significantly correlated (*r* = −.09, *N* = 43, *p* = .566), reflecting the uniqueness of these two personal value constructs.

A

B

*Supplementary Figure 2.* Mean log-transformed response times (log(RT)) over probability levels (A) collapsed across trial decision types and (B) separately for accepted and rejected trials. LL = Low-Low; ML = Middle-Low; MM = Middle-Middle; MH = Middle-High; HH = High-High. Error bars represent ±1 SE. The text beside each dot indicates the number of responses within each condition. Missed decisions (mean (SD) = 2.37 (3.34) per person) were not included in the figure.

*Supplementary Figure 3.* Associations between security and Lottery Choice Task (LCT) log-transformed response times (log(RT)). Participants’ mean log(RT) are shown over five levels of winning probability for acceptance (see Supplementary Table 1) and rejection response based on a median split of the security ratings (see Supplementary Figure 1). LL = Low-Low; ML = Middle-Low; MM = Middle-Middle; MH = Middle-High; HH = High-High. Error bars represent ±1 SE. The text beside each dot indicates the number of responses within each condition. Missed decisions (mean (SD) = 2.37 (3.34) per person) were not included in the figure. See Supplementary figure 5 for estimated log(RT) based on coefficients estimated using Equation 2.

*Supplementary Figure 4.* The estimated ARs modeled over winning probability based on Equation 1 and Supplementary Table 2 coefficients over three discrete magnitude levels (using the mean value of each magnitude level) and for high and low (A) hedonism or (B) security groups (using mean ratings of each group). Shaded bands indicate 95% confidence intervals. The dotted line marks the trend of a hypothetical rational decision-maker who solely relies on the expected value of lottery trials.

*Supplementary Figure 5.* The estimated log(RT) over winning probability based on the linear mixed model (see Equation 2 and Supplementary Table 3) for accepted and rejected stakes for high and low security groups (using mean ratings of each group). Shaded bands represent 95% confidence intervals.

*Supplementary Figure 6.* Simple slope analyses visualizing (A) the negative and (B) the positive personal value effects on neural sensitivity to the interactive effect of probability and magnitude. Parameter estimates are of conditional neural sensitivity to probability (CONDPROB; see Methods) derived for the three different magnitude levels. With decreasing magnitude level, individuals with higher hedonism reduced neural sensitivity to losing probability (negative neural sensitivity to winning probability); while individuals with higher security increased neural sensitivity to losing probability. ROI details are shown in Supplementary Tables 4 and 5, and Figures 3 and 4B. Shaded bands represent 95% confidence intervals. H = high; M = middle; L = low; L = left; R = right; DLPFC = dorsolateral prefrontal cortex; SOG = superior occipital gyrus.

References

Brosch, T., Coppin, G., Scherer, K. R., Schwartz, S., & Sander, D. (2011). Generating value(s): Psychological value hierarchies reflect context-dependent sensitivity of the reward system. *Social Neuroscience, 6*(2), 198-208. doi:10.1080/17470919.2010.506754

Buckner, R. L., Sepulcre, J., Talukdar, T., Krienen, F. M., Liu, H., Hedden, T., . . . Johnson, K. A. (2009). Cortical hubs revealed by intrinsic functional connectivity: Mapping, assessment of stability, and relation to Alzheimer's disease. *The Journal of Neuroscience, 29*(6), 1860-1873. doi:10.1523/jneurosci.5062-08.2009

Button, K. S., Ioannidis, J. P. A., Mokrysz, C., Nosek, B. A., Flint, J., Robinson, E. S. J., & Munafò, M. R. (2013). Power failure: Why small sample size undermines the reliability of neuroscience. *Nature reviews neuroscience, 14*(5), 365-376. doi:10.1038/nrn3475

Cox, R. W., Chen, G., Glen, D. R., Reynolds, R. C., & Taylor, P. A. (2017). Fmri clustering in afni: False-positive rates redux. *Brain Connectivity, 7*(3), 152-171. doi:10.1089/brain.2016.0475

Faul, F., Erdfelder, E., Lang, A.-G., & Buchner, A. (2007). G*power 3: A flexible statistical power analysis program for the social, behavioral, and biomedical sciences. *Behavior Research Methods, 39*(2), 175-191. doi:10.3758/BF03193146

Geuter, S., Qi, G., Welsh, R. C., Wager, T. D., & Lindquist, M. A. (2018). Effect size and power in fMRI group analysis. *bioRxiv*, 295048. doi:10.1101/295048

Goh, J. O. S., Su, Y.-S., Tang, Y.-J., McCarrey, A. C., Tereshchenko, A., Elkins, W., & Resnick, S. M. (2016). Frontal, striatal, and medial temporal sensitivity to value distinguishes risk-taking from risk-aversive older adults during decision making. *Journal of Neuroscience, 36*(49), 12498-12509. doi:10.1523/JNEUROSCI.1386-16.2016

Jenkinson, M., Beckmann, C. F., Behrens, T. E. J., Woolrich, M. W., & Smith, S. M. (2012). FSL. *Neuroimage, 62*(2), 782-790. doi:10.1016/j.neuroimage.2011.09.015

Kuss, K., Falk, A., Trautner, P., Montag, C., Weber, B., & Fliessbach, K. (2015). Neuronal correlates of social decision making are influenced by social value orientation—an fMRI study. *Frontiers in Behavioral Neuroscience, 9*(40). doi:10.3389/fnbeh.2015.00040

Maugeri, L., Moraschi, M., Summers, P., Favilla, S., Mascali, D., Cedola, A., . . . Fratini, M. (2018). Assessing denoising strategies to increase signal to noise ratio in spinal cord and in brain cortical and subcortical regions. *Journal of Instrumentation, 13*(02), C02028-C02028. doi:10.1088/1748-0221/13/02/c02028

Mumford, J. A., Poline, J.-B., & Poldrack, R. A. (2015). Orthogonalization of regressors in fMRI models. *PloS one, 10*(4), e0126255. doi:10.1371/journal.pone.0126255

Noble, S., Scheinost, D., & Constable, R. T. (2020). Cluster failure or power failure? Evaluating sensitivity in cluster-level inference. *Neuroimage, 209*, 116468. doi:<https://doi.org/10.1016/j.neuroimage.2019.116468>

Su, Y.-S., Chen, J.-T., Tang, Y.-J., Yuan, S.-Y., McCarrey, A. C., & Goh, J. O. S. (2018). Age-related differences in striatal, medial temporal, and frontal involvement during value-based decision processing. *Neurobiology of Aging, 69*, 185-198. doi:10.1016/j.neurobiolaging.2018.05.019

Sul, S., Tobler, P. N., Hein, G., Leiberg, S., Jung, D., Fehr, E., & Kim, H. (2015). Spatial gradient in value representation along the medial prefrontal cortex reflects individual differences in prosociality. *Proceedings of the National Academy of Sciences, 112*(25), 7851-7856. doi:10.1073/pnas.1423895112
